# Preference-based Health status in a German outpatient cohort with multiple sclerosis

**DOI:** 10.1186/1477-7525-11-162

**Published:** 2013-10-03

**Authors:** Jens Peter Reese, Gabriele Wienemann, Axel John, Alexandra Linnemann, Monika Balzer-Geldsetzer, Ulrich Otto Mueller, Christian Eienbröker, Björn Tackenberg, Richard Dodel

**Affiliations:** 1 Department of Neurology, Philipps-University, Marburg, Germany; 2 Institute of Medical Sociology and Social Medicine, Philipps-University, Marburg, Germany

**Keywords:** Health status, FAMS, EuroQol, Multiple sclerosis, Germany, Depression, Fatigue, Quality of life

## Abstract

**Background:**

To prospectively determine health status and health utility and its predictors in patients with multiple sclerosis (MS).

**Methods:**

A total of 144 MS patients (mean age: 41.0 ±11.3y) with different subtypes (patterns of progression) and severities of MS were recruited in an outpatient university clinic in Germany. Patients completed a questionnaire at baseline (n = 144), 6 months (n = 65) and 12 months (n = 55). Health utilities were assessed using the EuroQol instrument (EQ-5D, EQ VAS). Health status was assessed by several scales (Expanded Disability Severity Scale (EDSS), Modified Fatigue Impact Scale (M-FIS), Functional Assessment of MS (FAMS), Beck Depression Inventory (BDI-II) and Multiple Sclerosis Functional Composite (MSFC)). Additionally, demographic and socioeconomic parameters were assessed. Multivariate linear and logistic regressions were applied to reveal independent predictors of health status.

**Results:**

Health status is substantially diminished in MS patients and the EQ VAS was considerably lower than that of the general German population. No significant change in health-status parameters was observed over a 12-months period. Multivariate analyses revealed M-FIS, BDI-II, MSFC, and EDSS to be significant predictors of reduced health status. Socioeconomic and socio-demographic parameters such as working status, family status, number of household inhabitants, age, and gender did not prove significant in multivariate analyses.

**Conclusion:**

MS considerably impairs patients’ health status. Guidelines aiming to improve self-reported health status should include treatment options for depression and fatigue. Physicians should be aware of depression and fatigue as co-morbidities. Future studies should consider the minimal clinical difference when health status is a primary outcome.

## Introduction

Multiple sclerosis (MS) is a demyelinating autoimmune disease and a common cause of severe neurological disability in young adults in the Western world, presenting with diverse and often unpredictable symptoms and an uncertain course of progression. According to recent estimates, 1.3 million patients suffer from MS worldwide with 0.08-0.13% of the German population being affected [1-3].

The subtypes are defined by their pattern of progression; approximately 10-20% of MS patients suffer from primary progressive disease (PPMS), which has a slightly later onset compared to the other subtypes. The long-term prognosis remains generally poor; 15 years after diagnosis, over 80% of patients have functional and/or cognitive limitations and 50-60% require assistance to walk.

Health status (HS) in MS has been intensively studied in the past two decades and approximately 20 disease-specific health- status measures have been developed [4]. Predictors of health status reveal that physical and psychological symptoms are interrelated and that both are important. Depression is one of the strongest psychiatric predictors of poor health, as well as cognitive impairment and fatigue (for a review see e.g. [5]).

Psychological and behavioural symptoms have long been disregarded and undertreated in patients with MS [6]. The burden of living with MS affects physical and mental health and has negative effects on work life [7]. Neuropsychiatric symptoms such as fatigue present early in the course of disease, and specific cognitive impairments can be observed in more than half of patients in the early stages [8,9]. Even in patients with short (less than 2 years) disease durations, discrete impairment of cognitive function may be observed in up to 60% of patients upon neuropsychological testing [10]. Symptoms of depression also present early in the course of disease and affect cognitive performance. Within a year of diagnosis, 48% of patients and 46% of their relatives show clinically relevant anxiety, depression or distress [11,12].

Therapies that are newly available or undergoing clinical trials may place an economic strain on health-care systems. Therefore, one important aspect in the appraisal of new treatments is the effectiveness at improving health status.

The aim of this study was to systematically explore factors associated with MS and their contribution to decreases in patient-reported health status. To address this question, we used a longitudinal study design in which patients were repeatedly questioned over a 12-month period. We used the EuroQoL (EQ-5D) instrument and its Visual Analogue Scale (EQ VAS) [13] as well as the disease-specific Functional Assessment of MS (FAMS) [14] to quantify preference-based measures.

## Patients and methods

### Patient selection

Patients were recruited in the Department of Neurology, Philipps-University Marburg from October to December 2007 for baseline assessments. A convenience sample was recruited from 186 patients admitted to the Department of Neurology during that time. 77.4% of these patients participated. Patients were included if they fulfilled the McDonald diagnostic criteria for MS [15,16]. The case report form (CRF) consisted of two parts: one to be completed by the examining physician and one to be completed by the patient. Patients could complete their part of the CRF during their hospital stay or return it later by mail. Patients were assessed three times: at baseline (n = 144) and at follow-up assessments after 6 (n = 65) and 12 months (n = 55). The local ethics committee of Philipps-University Marburg approved this study and all patients gave written consent prior to participation.

### Measurements

The questionnaire consisted of an evaluation of the socio-demographic, clinical, and health status of patients suffering from MS. In addition, health-care resource utilisation for MS and indirect costs were examined.

In the patients’ part of the CRF, health status was first evaluated by an MS-specific measure: the Functional Assessment of MS (FAMS) which consists of four subscales covering mobility (“I have trouble walking”), symptoms (“I have pain”), emotional well-being (“I am able to enjoy life”), and general contentment (“I have accepted my illness”) with seven items each and scores ranging from 0 (poor QoL) to 28 (high QoL), as well as a thinking-and-fatigue subscale (“I feel tired”) that contains 8 items and is scored from 0 to 36. The FAMS sum score has a maximum score of 172 [14].

Second, health status was measured by the generic EuroQoL instrument. Applicable to a wide range of health conditions and treatments, EQ-5D generates a health profile, but is also capable of expressing health status as a single index value (utility value). Thus, it can be used for clinical and economical evaluations of health care. The questionnaire consists of five questions representing five different dimensions (“Mobility”, “Self-Care”, “Usual Activities”, “Pain/Discomfort”, and “Anxiety/Depression”). An index score can be derived by time-trade-off methods based on a recent European study [13]. The second part of the EuroQol instrument consists of a visual analogue scale (EQ VAS) on which respondents rate their subjective health status on a scale from 0 (poorest) to 100 (best imaginable) [13].

The impact of fatigue was assessed using the Modified Fatigue Impact Scale (M-FIS) [17]. The scale consists of 21 items, with 10 items related to mental fatigue and 11 items related to physical and social fatigue. The scoring ranges between 0 and 82, with higher scores reflecting greater impact. In the literature, a cut-off value of 38 has been used to distinguish fatigued from non-fatigued patients [18,19].

In addition, depression was assessed using the Beck Depression Inventory (BDI-II), a 21-item scale used to measure self-reported severity of depression. According to common conventions, the BDI sum score was categorised into five groups: no (0–8 points), minimal (9–13 points), mild (14–19 points), moderate (20–28 points), and severe (>28 points) depressive symptoms [20,21].

The second part of the CRF was to be completed by the examining physician and concerned the pattern of progression (MS subtype), comorbidities, neuropsychiatric symptoms, and the assessment of disease severity by the Expanded Disability Status Scale (EDSS) [22] and the MS Functional Composite (MSFC) [23].

The EDSS is an MS-specific scale consisting of a neurological assessment quantifying disability in eight functional systems (e.g., sensory functions, cerebellar functions). The assessment of these functional systems yields a sum score ranging from 0 (no neurological impairment) to 10 (death due to MS) [24].

The MS Functional Composite (MSFC) comprises quantitative functional measures of three key clinical dimensions of MS: leg function/ambulation, arm/hand function, and cognitive function. Scores on component measures are converted to standard scores (z-scores), which are averaged to yield a single MSFC score [25].

### Statistical analysis

Results are presented as means ± SD and median or percentages as appropriate. Groups were compared by the appropriate parametric and nonparametric tests (*t*-test, Mann–Whitney-U test, Kruskall-Wallis test). The baseline investigation and the follow-up investigations were compared using repeated-measures analysis of variance. Missing values were handled by pairwise deletion. The potential changes in the five EQ-5D domains were analysed using univariate repeated-measurement tests. Variables were screened for impact on health status using the Spearman rank correlation (p = 0.10). From the variables that proved significant in bivariate analyses, reasonable variables were chosen for multiple linear regression with EQ VAS, EQ-5D index score, and FAMS sum score as dependent variables. We used a stepwise forward mode to include independent predictors with a significance level of α=0.05. Uni- and multivariate logistic regression analyses were applied to test for significant predictors of single dimensions of the EQ-5D index. The variables for the five dimensions were dichotomised into “having problems” or “having no problems” (e.g., “no problems with daily activities” was represented as 0, “some problems” or “severe problems with daily activities” was represented as 1). Statistical analyses were performed with the SPSS 19.0 software package (IBM Inc., Armonk, NY.).

## Results

### Clinical and socio-demographic characteristics

A total of 144 MS patients were evaluated in this study: 99 women (68.3%) and 45 men (31.7%). At the 6-month follow-up, 65 patients remained in the study; at the 12-month follow-up, 55 patients remained. Furthermore, 7 patients did not complete the demographic questionnaire at baseline and were therefore excluded from further analyses. The patients’ demographic and clinical data are presented in Table 1.

**Table 1:** Demographic and clinical data of the study sample at baseline (n = 137)

**Variable**	**RRMS**	**SPMS**	**PPMS**	**Total**
	**% (n)**	**% (n)**	**% (n)**	**% n**
**Females % (n)**	**67.7 (63)**	**29.1 (27)**	**3.2 (3)**	**100 (93)**
**Males % (n)**	**65.9 (29)**	**25.0 (11)**	**9.1 (4)**	**100 (44)**
	**Mean (SD)**	**Mean (SD)**	**Mean (SD)**	**Mean (SD )**
Age (years)	36.4 (9.7)	50.4 (8.9)	50.5 (10.4)	41.1 (11.3)
Disease duration (years)	5.3 (5.1)	13.6 (8.1)	8.3 (11.7)	7.3 (7.2)
MSFC Z-composite score	0.100 (0.632)	−0.670 (0.654)	−0.882 (1.128)	- 0.121 (0.750)
M-FIS sum score	33.2 (20.4)	44.1 (13.7)	40.6 (15.2)	36.4 (18.6)
FAMS sum score	120.3 (33.2)	101.7 (26.3)	98.1 (27.6)	113.3 (32.4)
EQ-5D index score	0.83 (0.18)	0.64 (2.78)	0.64 (0.26)	0.77 (0.23)
EQ VAS	68.4 (18.2)	50.6 (18.4)	44.8 (20.1)	62.0 (20.5)
BDI sum score	14.7 (10.2)	14.5 (8.6)	16.2 (11.2)	14.7 (10.4)
EDSS score	2.6 (1.5)	5.4 (1.5)	5.9 (1.2)	3.5 (2.0)

MSFC: Multiple Sclerosis Functional Composite, M-FIS: Modified Fatigue Impact Scale, FAMS: Functional Assessment in Multiple Sclerosis, EQ-5D: EuroQoL, EQ VAS: Visual Analogue Scale, BDI: Beck Depression Inventory II, EDSS: Extended Disability Symptom Scale; SD: standard deviation.

Most of the patients lived with partners (76.1%); 45% had a secondary school education (or higher); and 38% stated that their career advancement was impaired by MS. In addition, 37% claimed that due to the disease, they earned less than their unaffected colleagues.

### Health status

At baseline, the distribution of patients among the different subtypes of MS was as follows: 66.8% relapsing-remitting multiple sclerosis (RRMS), 27% secondary progressive multiple sclerosis (SPMS), and 6.2% primary progressive multiple sclerosis (PPMS). Within the 12-months observation period, the median EDSS score increased from 3.50 (range 0 to 8.5) to 4.00 (range 0 to 9.00) with 60.3% of patients progressing to higher EDSS stages. The mean total MSFC z-score improved minimally, from −0.100 (SD: 0.741) to 0.199 (SD: 0.682) during the 12-month observation period (Table 2). SPMS and PPMS patients were older and had worse disability scores (EDSS and MSFC, Table 1) than RRMS patients at baseline.

**Table 2:** Clinical characteristics of patients with multiple sclerosis included in this study and results of EDSS, EQ-5D, FAMS, MSFC, M-FIS, and BDI-II at baseline, 6 months and 12 months follow-up investigation

**Variable**	**Baseline**	**6 months**	**Δ Baseline – 6 M**	**12 months**	**Δ Baseline – 12 M**
	**Median (range)**	**Median (range)**	***p*-value**	**Median (range)**	***p*-value**
	**Mean ± SD**	**Mean ± SD**	***p*-value**	**Mean ± SD**	***p*-value**
**EDSS** (N = 55)	3.50 (0–8.5)	3.25 (0–8.0)	n.s.	4.0 (0–9.0)	<0.001
**EuroQol**					
EQ-5D Index Score (N = 56)	0.79 ±0.20	0.79 ±0.19	n.s.	0.78 ±0.23	n.s.
EQ VAS (N = 51)	59.84 ±20.0	61.82 ±18.7	n.s.	61.37 ±18.4	n.s.
**FAMS** (N = 56)					
Sum Score	113.7 ±32.3	115.1 ± 31.6	n.s.	110.3 ± 31.6	n.s.
Mobility	16.7 ±7.1	16.4 ± 7.0	n.s.	15.7 ± 7.1	n.s.
Symptoms	20.1 ±5.6	20.3 ± 5.3	n.s.	20.1 ± 5.2	n.s.
Emotional Well-being	19.3 ±6.2	19.8 ± 6.0	n.s.	18.9 ± 6.1	n.s.
General Contentment	17.2 ±6.6	17.5 ± 6.2	n.s.	16.8 ± 5.9	n.s.
Thinking and Fatigue	21.0 ±9.1	21.4 ± 9.2	n.s.	19.3 ± 9.6	n.s.
Family/ Social Well-being	19.5 ±6.5	19.7 ± 6.5	n.s.	19.5 ± 5.4	n.s.
**MSFC (Z-composite)** (N = 55)	- 0.162 ±0.764	−0.155 ± 0.763	n.s.	- 0.120 ±0.985	n.s.
**M-FIS Sum Score** (N = 52)	37.9 ±18.1	37.4 ±21.4	n.s.	36.1 ±20.6	n.s.
**BDI Sum Score** (N = 55)	14.4 ±9.8	14.8 ±10.0	n.s.	14.6 ±8.9	n.s.
	**% (n)**	**% (n)**		**% (n)**	
Normal (0–8 pts)	30.9 (17)	32.7 (18)		25.5 (14)	
Minimal (9–13 pts)	27.3 (15)	21.8 (12)		14.5 (8)	
Mild (14–19 pts)	20.0 (11)	23.7 (13)		21.8 (12)	
Medium (20–28 pts)	14.5 (8)	10.9 (6)		32.7 (18)	
Severe (29–63 pts)	7.3 (4)	10.9 (6)		5.5 (3)	

EDSS Extended Disability Symptom Scale, EQ-5D EuroQoL, EQ VAS Visual Analogue Scale, FAMS Functional Assessment in Multiple Sclerosis, MSFC Multiple Sclerosis Functional Composite, M-FIS Modified Fatigue Impact Scale, BDI-II Beck Depression Inventory.

Changes from baseline to either 6 months or 12 months did not reach statistical significance (p > 0.10, Pillai’s Trace) except for EDSS at 12 months.

The PPMS group was the most impaired group by fatigue and depression. Clinically relevant depressive symptoms, as measured by BDI-II, were very common in the study population (Table 2). In all, 61 patients (41.5%) had depressive symptoms (BDI sum score >13pts); 12 (8.2%) thereof had severe depressive symptoms at baseline. Depressive symptoms were significantly positively correlated with Expanded Disability Status (Jonckheere-Terpstra J = 5163, z = 284.7, r = 23.7, *p* = 0.04). High BDI scores were also correlated with low MSFC z-scores indicating that disease severity is associated with depressive symptoms (*p* = 0.032, r = −0.190).

PASAT scores (below the 5^th^ percentile) revealed 71 patients (49.3%) with cognitive impairment with no significant difference between genders. A total of 24 (16.7%) patients had pain syndromes, and 4.2% suffered from anxiety.

Fatigue was also a common symptom, reported by 63.2% (n = 91) of the patients. The mean score of the M-FIS at baseline was 36.5 (SD: 18.5).

Patient-reported health status was assessed by the MS-specific FAMS and the generic EuroQol instrument. The mean total score of the FAMS was 113 (SD: 34) at baseline. The FAMS sum score and all of its sub-scores except family/social well-being were significantly positively correlated with disease severity, as measured by MSFC.

At the 12-month follow-up, no significant change was observed in the FAMS sum score or in any of its subscales.

**Figure 1:**
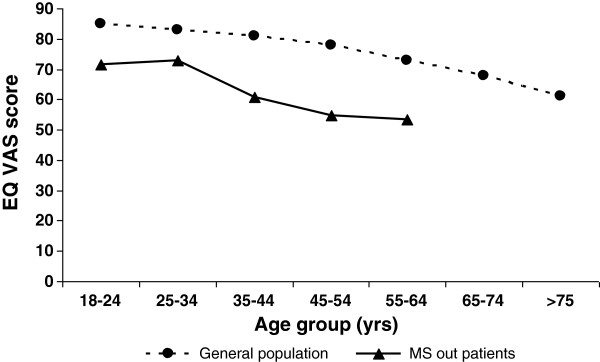
**Mean EQ VAS score for age-grouped MS patients ranking their health status using the EQ VAS compared to a representative sample of the German general population**[26]**.**

**Figure 2:**
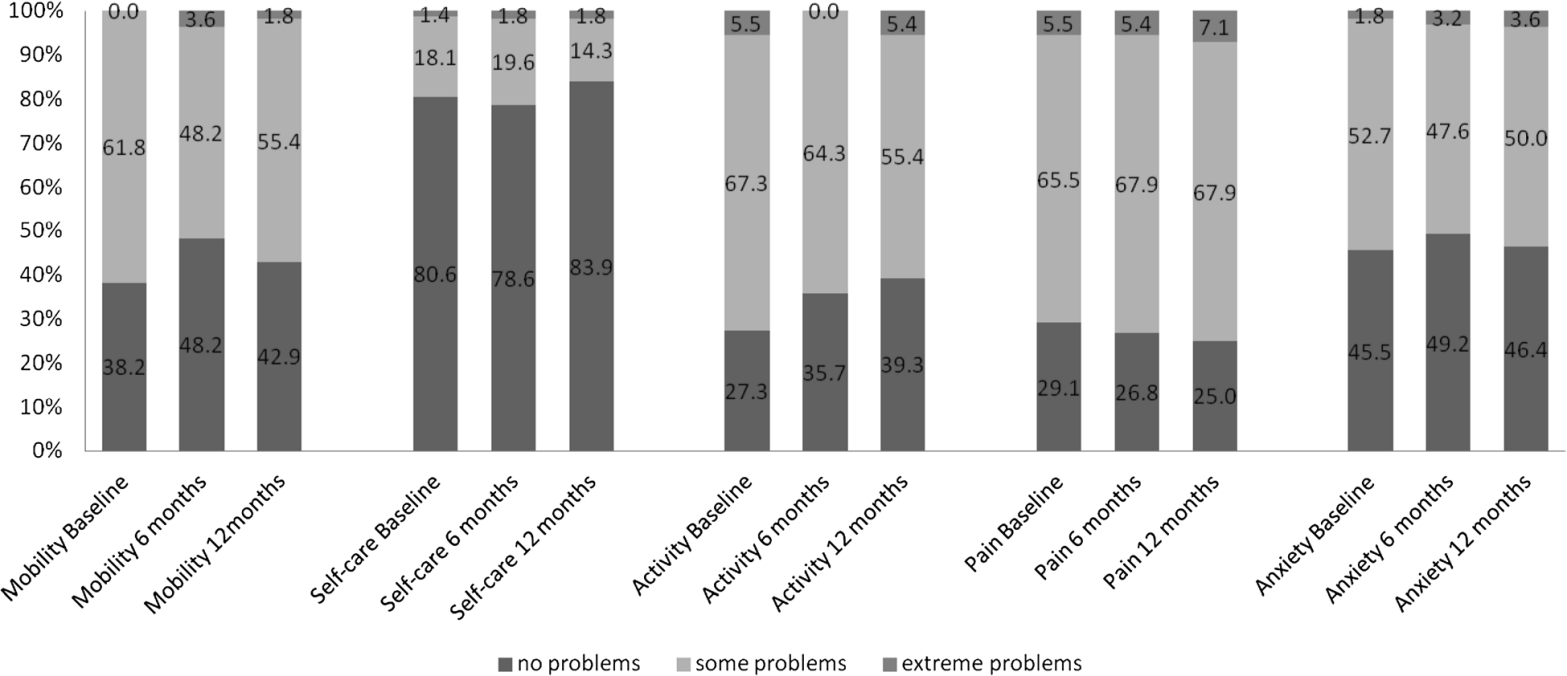
**Quality of life parameters from baseline to month 6 and month 12 (subjective assessment of the EQ-5D domains at baseline and after 6 and 12 months (n = 55)).** Changes from baseline to either 6 months or 12 months did not reach statistical significance (*p* > 0.18, Pillai’s Trace).

The EQ-5D index score was 0.77 (SD: 0.23) at baseline. The mean score on the EQ VAS was 62.0 (SD: 20). The EQ VAS of MS patients differed considerably from that of the general population, as depicted in Figure 1. Neither measure showed any change after one year (Figure 2).

We also analysed negative effects on the dimensions measured by the EQ-5D questionnaire (Table 3, Figure 2). In each dimension, MS patients reported problems more frequently than the general population. Approximately 60% reported problems with mobility (vs. 16% in the general population); 20% had problems with self-care (vs. 3%); 65% had problems with usual activities (vs. 10%); 70% suffered from pain (vs. 28%); and 56% had problems with anxiety and/or depression. No significant difference between genders was observed, except in self-care: 23% of male MS patients had problems with self-care, whereas only 14% of female patients reported such problems. Furthermore, we used univariate and multivariate analyses to determine whether demographic data such as disease subtype, age, and/or gender predict reported impairments in the EQ-5D dimensions. RRMS, SPMS, and age were significant predictors of problems with mobility, self-care, and usual activities. RRMS and SPMS were also significant predictors of problems with anxiety or depression, but none of the demographic variables predicted problems with the EQ-5D dimension “pain”. Gender was significantly correlated with problems in the dimension “usual activities”.

**Table 3:** Percentage of age-grouped individuals with problems in the different EQ-5D dimensions

	**Mobility**			**Self-care**			**Usual activities**			**Pain**			**Anxiety/depression**		
	**Tot.**	**M**	**F**	**Tot.**	**M**	**F**	**Tot.**	**M**	**F**	**Tot.**	**M**	**F**	**Tot.**	**M**	**F**
**Gen. Pop.**^**1 **^**[%]**	**15.9**	**14.9**	**16.8**	**2.8**	**2.2**	**3.4**	**9.9**	**8.9**	**10.7**	**27.6**	**24.9**	**30.0**	**4-3**	**3.6**	**4.9**
**MS**^**2 **^**[%]**	**60.4**	**55.6**	**62.6**	**19.4**	**22.2**	**18.2**	**65.3**	**62.2**	**66.7**	**69.4**	**62.2**	**72.7**	**56.3**	**44.4**	**61.6**
	**UV OR [CI]**	**MV OR [CI]**		**UV OR [CI]**	**MV OR [CI]**		**UV OR [CI]**	**MV OR [CI]**		**UV OR [CI]**	**MV OR [CI]**		**UV OR [CI]**	**MV OR [CI]**	
RRMS^§^	.077** [.026;.232]	n.s.		.07** [.04;.25]	n.s.		.10** [.03;.31]	n.s.		n.s.	n.s.		n.s.	17.92* [1.50;214.83]	
SPMS	8.92** [2.96;26.87]	n.s.		8.14** [3.29;20.15]	n.s.		9.72** [2.82;33.57]	n.s.		n.s.	n.s.		n.s.	22.83* [1.94;269.30]	
Age	1.11** [1.07;1.15]	n.s.		1.11** [1.06;1.16]	n.s.		1.10** [1.03;1.10]	n.s.		n.s.	n.s.		n.s.	n.s.	
Gender	n.s.	n.s.		n.s.	n.s.		.82** [.40;1.72]	n.s.		n.s.	n.s.		n.s.	n.s.	
Disease Duration	1.16** [1.08;1.25]	n.s.		1.14** [1.07;1.21]	n.s.		1.13* [1.05;1.21]	n.s.		n.s.	n.s.		n.s.	n.s.	
EDSS Score	3.19** [2.20;4.61]	2.86** [1.95;4.18]		4.01** [2.37;6.81]	4.10** [2.36;7.12]		1.94** [1.50;2.50]	1.61* [1.21;2.13]		n.s.	n.s.		n.s.	n.s.	
MSFC z-score ^§^	.19** [.09;.37]	n.s.		.08** [.03;.23]	n.s.		.31** [.17;.58]	n.s.		n.s.	n.s.		n.s.	n.s.	
BDI Score	n.s.	n.s.		n.s.	n.s.		1.10** [1.05;1.15]	n.s.		1.14** [1.07;1.21]	n.s.		1.19** [1.12;1.28]	1.13* [1.04;1.24]	
M-FIS Score	1.05** [1.03;1.08]	n.s.		1.04* [1.02;1.07]	n.s.		1.08** [1.05;1.10]	n.s.		1.08** [1.05;1.11]	1.08** [1.05;1.12]		1.06 ** [1.04;1.09]	n.s.	
FAMS Score^§^	.97** [.96;.99]	n.s.		.98* [.96;.99]	n.s.		.96** [.94;.97]	.96** [.95;.98]		.96** [.95;.98]	n.s.		.96** [.95;.98]	.97* [.05;.99]	

^1,2^Percentage of people having problems in the EQ-5D subdimensions: comparison between data concerning the general population [26] and the specific MS population in our study, ^§^protecting factors, **(p < .001); *(p < .05), n.s. not significant (p > .05).

RRMS Relapsing-Remitting MS; SPMS Secondary Progressive MS; EDSS Extended Disability Symptom Scale, MSFC Multiple Sclerosis Functional Composite; BDI-II Beck Depression Inventory; M-FIS Modified Fatigue Impact Scale; FAMS Functional Assessment of Multiple Sclerosis; Gen. Pop.: General population; Tot.: Total; M: male; F: female.

Uni- and multivariate logistic regression analysis identifying predictors for each EQ-5D subdimension.

Next, the impact of clinical symptoms and socioeconomic variables on health status were analysed in multivariate regression analyses (Table 4). The regression models fit the data well to identify predictor of health status in terms of FAMS and EQ-5D. M-FIS, BDI-II, MSFC and EDSS were identified as significant predictors in multivariate analyses. Higher scores in M-FIS, BDI-II, and EDSS predicted lower scores in FAMS and EQ-5D while lower scores in MFSC predicted lower scores in FAMS and EQ-5D. Socioeconomic and socio-demographic parameters such as working status, family status, number of household inhabitants, age, and gender did not prove significant in multivariate analyses.

**Table 4:** Analysis of predictor variables for the FAMS score, EQ-5D index score and EQ VAS

	**FAMS score**			**EQ-5D index score**			**EQ VAS**		
	beta	95% CI for B	*p*-value	beta	95% CI for B	*p*-value	beta	95% CI for B	*p*-value
Constant		157.067;191.542	<0.001		0.851;1.291	<0.001		65.058;101.797	<0.001
M-FIS	−0.611	−1.289;-0.831	<0.001	−0,431	−0.008;-0.002	<0.001	−0.523	−0.809;-0.325	<0.001
BDI-II	−0.344	−1.579;-0.759	<0.001	−0,040	−0.006;0.004	n.s.	−0.094	−0.638;0.237	n.s.
MSFC (z-score)	0.104	0.480;8.680	<0.05	0,194	0.004;0.109	<0.05	0.268	3.002;11.666	0.001
EDSS	−0.209	−6.129;1.411	n.s.	−0.259	−0.063;-0.001	<0.05	−0.281	−5.617;-0.670	<0.05
Age	0.018	−0.251;0.360	n.s.	−0.044	−0.005;0.003	n.s.	0.035	−0.263;0.393	n.s.
Gender	−0.060	−10.103;1.625	n.s.	−0.030	−0.88;0.060	n.s.	−0.007	−6.478;5.888	n.s.
Working status	−0.021	−2.166;1.325	n.s.	−0.026	−0.026;0.019	n.s.	−0.032	−2.269;1.470	n.s.
Family status	0.020	−2.221;3.607	n.s.	−0.046	−0.048;0.026	n.s.	0.081	−1.312;4.813	n.s.
Number of household inhabitans	0.033	−0.167;0.394	n.s.	0.134	0.000;0.007	n.s.	0.101	−0.077;0.512	n.s.
		R^2^ = 0.840			R^2^ = 0.420			R^2^ = 0.581	

beta: Beta standardized linear coefficient, 95% CI: 95% confidence interval for unstandardized linear coefficients, R2: coefficient of determination.

FAMS Functional Assessment in Multiple Sclerosis; EQ-5D EuroQoL, EQ VAS Visual Analogue Scale, M-FIS Modified Fatigue Impact Scale, BDI-II Beck Depression Inventory, MSFC: Multiple Sclerosis Functional Composite.

## Discussion

This prospective study evaluated the impact of MS on health status using the generic EuroQol instrument and the disease-specific measure FAMS. In line with previous studies, we have shown that MS substantially affects health status (Figure 1; for a review, see [5]).

Many health-status assessments have focused on MS patients, but our study aimed to address three particular gaps. First, only few German studies have assessed patient preferences to derive QALY measures for economic evaluation [27,28]. Second, health-status assessments stratified by MS subtype are scarce; here, we provide insight into the health- status differences associated with the three different types of MS (RRMS, SPMS, PPMS) in a well-characterised patient population. Third and most importantly, we provide a detailed longitudinal analysis of patient-relevant outcomes in MS.

Concerning problems in the EQ-5D domains, we show considerable impairments in health status in our study population compared to the general German population. Our results, however, differ from those found in the study by Putzki et al., as our cohort reported more problems. In our study, approximately 60% of MS patients reported some or extreme problems with mobility (vs. 38% in the study by Putzki et al. [28]); 71% had problems with usual activities (vs. 42%); 69% suffered from pain (vs. 50%); 56% reported problems with anxiety and depression (vs. 52%); and 17% had problems with self-care (vs. 9%). Although the patients in our study sample reported problems in the five dimensions more frequently, these problems were most likely less extreme, explaining the very similar mean index scores in the two studies.

Furthermore, the influence of psychological symptoms such as depression and fatigue on self-perceived health status was assessed. The multivariate linear and logistic regression demonstrated the importance of depression and fatigue to self-perceived health status. Previous studies also identified those variables as independent predictors of health-related quality of life [29,30]. Two studies have identified depression as the strongest determinant of impaired quality of life in patients with MS [31,32]. Recent studies relate symptoms of fatigue, major contributor to the burden of disease, to sleep disorders [33-36], thus emphasising the necessity of considering non-disease-specific symptoms when treating patients with MS. Depression and fatigue are important facets of MS and must be considered not as secondary outcomes, but as an integral part of disease presentation and management.

**Figure 3:**
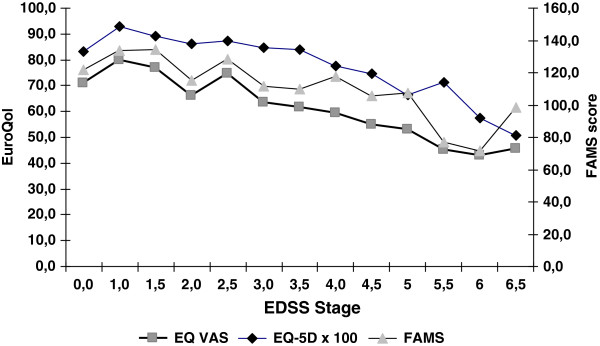
Mean health utility (EQ-5D, EQ VAS) and health status (FAMS) scores for MS patients by EDSS points.

We also confirm a strong correlation between health status and disability level (measured by EDSS), as shown in recent publications (for a review, see [5]) in Germany [27]. As illustrated in Figure 3, FAMS and health-utility measure EuroQol may not adequately distinguish among the different degrees of disease severity (i.e., the EDSS groups) and may therefore be inappropriate outcome measures for clinical and economic studies. Thus, we also applied the MSFC, which shows better scaling properties in combination with health-utility measures [37,38].

We could not demonstrate any change in health- status measurements over a 12 month observation period in our cohort, although the EDSS changed significantly during this time. Therefore, studies evaluating patient-reported outcomes using the EQ-5D and FAMS should encompass longer observation periods. Recently, a large German study (n = 674) compared EuroQoL health status before and 12 months following beta-interferon treatment and detected a difference of only 0.02 pts in the EQ-5D index score; this finding is significant but not clinically relevant [28]. The baseline value in that study (0.75) is similar to ours (0.77).

Our study has some limitations: First, the study is based on a convenience sample rather than an epidemiological survey, and only MS patients from a specialised university outpatient clinic were included. Therefore, generalisations should be made cautiously. The sample may be not representative of the German population of MS patients or MS patients of other geographic or ethnic origins. As patients were not evenly distributed across levels of care, more or less severely affected patients may bias the health-status results. Very few patients in our sample have disability levels higher than EDSS 6.5. Our results therefore apply only to patients in levels with less pronounced disabilities. Additionally, relapses may have a substantial influence on health status and self-reported quality of life in patients with MS [27,28]. We did not collect data on relapses and therefore cannot exclude the possibility that patient populations with different patterns of progression and relapse frequencies may show different results. Furthermore, quality of life is a multi-faceted phenomenon influenced by multiple other factors apart from those being measured in this study. For example, it was recently shown that certain patterns of visual impairment are specific to patients with MS [39,40] and considerably affect health-related quality of life [41]. Patients attach great importance to visual functioning, which should be taken into account when assessing patients with MS [42]. It is of utmost importance to comprehensively assess factors being related to quality of life.

In conclusion, this study is in line with previous findings and confirms that MS has a substantial impact on the self-perceived health status of the patients. Depression and fatigue are major predictors of impaired health status and should be considered integral parts of MS in disease management. We observed no significant changes after 6 or 12 months of observation in any patient-reported outcome used in this study. Our data may help in estimating sample sizes for future clinical trials using patient-reported outcomes.

## Conclusion

The quality of life and health status of patients with MS, as well as their correlation with clinical and socio-demographic outcomes, were comprehensively assessed in a longitudinal study over 12 months. In addition to an extensive and detailed clinical examination, a thorough evaluation of fatigue, depression, quality of life, and disease severity was included. MS considerably impairs patients’ health status, with depression and fatigue contributing considerably to the burden of disease. Guidelines aiming to improve self-reported health status should include treatment options for depression and fatigue. Physicians should be aware of depression, fatigue and other non-disease-specific parameters as comorbidities. Future studies should consider the minimal clinical difference when health status is a primary outcome.

## Competing interests

The authors declare that they have no competing interests.

## Authors’ contributions

JPR drafted the manuscript, participated in study design and conducted analysis. GW assessed the patients and participated data analysis. AJ assessed the patients and participated in data analysis. AL conducted data analysis and revised the manuscript. MBG planned study design, secured data quality and revised the manuscript. UOM revised the manuscript and data analysis. BT planned study design and revised the manuscript. RD planned study design, revised data analysis and the manuscript. All authors read and approved the final manuscript.

## References

[B1] Pugliatti M Rosati G Carton H Riise T Drulovic J Vecsei L Milanov I The epidemiology of multiple sclerosis in Europe Eur J Neurol 2006 13 700 722 10.1111/j.1468-1331.2006.01342.x 16834700

[B2] Pugliatti M Sotgiu S Rosati G The worldwide prevalence of multiple sclerosis Clin Neurol Neurosurg 2002 104 182 191 10.1016/S0303-8467(02)00036-7 12127652

[B3] WHO Atlas Multiple Sclerosis Resources in the World 2008 New York: WHO Library

[B4] Gruenewald DA Higginson IJ Vivat B Edmonds P Burman RE Quality of life measures for the palliative care of people severely affected by multiple sclerosis: a systematic review Mult Scler 2004 10 690 704 10.1191/1352458504ms1116rr 15584496

[B5] Mitchell AJ Benito-Leon J Gonzalez JM Rivera-Navarro J Quality of life and its assessment in multiple sclerosis: integrating physical and psychological components of wellbeing Lancet Neurol 2005 4 556 566 10.1016/S1474-4422(05)70166-6 16109362

[B6] Benedict RH Wahlig E Bakshi R Fishman I Munschauer F Zivadinov R Weinstock-Guttman B Predicting quality of life in multiple sclerosis: accounting for physical disability, fatigue, cognition, mood disorder, personality, and behavior change J Neurol Sci 2005 231 29 34 10.1016/j.jns.2004.12.009 15792817

[B7] Rivera-Navarro J Morales-Gonzalez JM Benito-Leon J Informal caregiving in multiple sclerosis patients: data from the Madrid Demyelinating Disease Group study Disabil Rehabil 2003 25 1057 1064 10.1080/0963828031000137766 12944155

[B8] Achiron A Barak Y Cognitive impairment in probable multiple sclerosis J Neurol Neurosurg Psychiatry 2003 74 443 446 10.1136/jnnp.74.4.443 12640060 PMC1738365

[B9] Brassington JC Marsh NV Neuropsychological aspects of multiple sclerosis Neuropsychol Rev 1998 8 43 77 10.1023/A:1025621700003 9658410

[B10] Ruggieri RM Palermo R Vitello G Gennuso M Settipani N Piccoli F Cognitive impairment in patients suffering from relapsing-remitting multiple sclerosis with EDSS < or = 3.5 Acta Neurol Scand 2003 108 323 326 10.1034/j.1600-0404.2003.00157.x 14616301

[B11] Janssens AC van Doorn PA de Boer JB van der Meche FG Passchier J Hintzen RQ Impact of recently diagnosed multiple sclerosis on quality of life, anxiety, depression and distress of patients and partners Acta Neurol Scand 2003 108 389 395 10.1034/j.1600-0404.2003.00166.x 14616290

[B12] Landro NI Celius EG Sletvold H Depressive symptoms account for deficient information processing speed but not for impaired working memory in early phase multiple sclerosis (MS) J Neurol Sci 2004 217 211 216 10.1016/j.jns.2003.10.012 14706226

[B13] Greiner W Claes C Busschbach JJ von der Schulenburg JM G Validating the EQ-5D with time trade off for the German population Eur J Health Econ 2005 6 2 124 130 10.1007/s10198-004-0264-z 19787848

[B14] Cella DF Dineen K Arnason B Reder A Webster KA Karabatsos G Chang C Lloyd S Steward J Stefoski D Validation of the functional assessment of multiple sclerosis quality of life instrument Neurology 1996 47 129 139 10.1212/WNL.47.1.129 8710066

[B15] Fangerau T Schimrigk S Haupts M Kaeder M Ahle G Brune N Klinkenberg K Kotterba S Mohring M Sindern E Diagnosis of multiple sclerosis: comparison of the Poser criteria and the new McDonald criteria Acta Neurol Scand 2004 109 385 389 10.1111/j.1600-0404.2004.00246.x 15147460

[B16] McDonald WI Compston A Edan G Goodkin D Hartung HP Lublin FD McFarland HF Paty DW Polman CH Reingold SC Recommended diagnostic criteria for multiple sclerosis: guidelines from the International Panel on the diagnosis of multiple sclerosis Ann Neurol 2001 50 121 127 10.1002/ana.1032 11456302

[B17] Fisk JD Ritvo PG Ross L Haase DA Marrie TJ Schlech WF Measuring the functional impact of fatigue: initial validation of the fatigue impact scale Clin Infect Dis 1994 18 Suppl 1 S79 S83 8148458 10.1093/clinids/18.supplement_1.s79

[B18] Flachenecker P Kumpfel T Kallmann B Gottschalk M Grauer O Rieckmann P Trenkwalder C Toyka KV Fatigue in multiple sclerosis: a comparison of different rating scales and correlation to clinical parameters Mult Scler 2002 8 523 526 10.1191/1352458502ms839oa 12474995

[B19] Tellez N Rio J Tintore M Nos C Galan I Montalban X Does the Modified Fatigue Impact Scale offer a more comprehensive assessment of fatigue in MS? Mult Scler 2005 11 198 202 10.1191/1352458505ms1148oa 15794395

[B20] Beck AT Steer RA Brown GK Beck Depression Inventory. 2nd edn 1996 San Antonio: TX: The Psycholigal Corporation

[B21] Hautzinger M Keller F Kühner C BDI-II. Beck-Depressions-Inventar. Revision 2009 Pearson Assessment: Frankfurt

[B22] Kurtzke JF Rating neurologic impairment in multiple sclerosis: An expanded disability status scale (EDSS) Neurology 1983 33 1444 10.1212/WNL.33.11.1444 6685237

[B23] Fischer JS Rudick RA Cutter GR Reingold SC Force NMSCOAT The Multiple Sclerosis Functional Composite measure (MSFC): an integrated approach to MS clinical outcome assessment Mult Scler 1999 5 244 250 10467383 10.1177/135245859900500409

[B24] Kurtzke JF Rating neurologic impairment in multiple sclerosis: an expanded disability status scale (EDSS) Neurology 1983 33 1444 1452 10.1212/WNL.33.11.1444 6685237

[B25] Fischer JS Rudick RA Cutter GR Reingold SC The Multiple Sclerosis Functional Composite Measure (MSFC): an integrated approach to MS clinical outcome assessment. National MS Society Clinical Outcomes Assessment Task Force Mult Scler 1999 5 244 250 10467383 10.1177/135245859900500409

[B26] Koenig HH Bernert S Angermeyer MC Health Status of the German population: results of a representative survey using the EuroQol questionnaire Gesundheitswesen 2005 67 173 182 10.1055/s-2005-857991 15789280

[B27] Kobelt G Berg J Lindgren P Elias WG Flachenecker P Freidel M Konig N Limmroth V Straube E Costs and quality of life of multiple sclerosis in Germany Eur J Health Econ 2006 7 Suppl 2 S34 S44 17310337 10.1007/s10198-006-0384-8

[B28] Putzki N Fischer J Gottwald K Reifschneider G Ries S Siever A Hoffmann F Kafferlein W Kausch U Liedtke M Quality of life in 1000 patients with early relapsing-remitting multiple sclerosis Eur J Neurol 2009 16 713 720 10.1111/j.1468-1331.2009.02572.x 19475754

[B29] Amato MP Ponziani G Rossi F Liedl CL Stefanile C Rossi L Quality of life in multiple sclerosis: the impact of depression, fatigue and disability Mult Scler 2001 7 340 344 11724451 10.1177/135245850100700511

[B30] Janardhan V Bakshi R Quality of life in patients with multiple sclerosis: the impact of fatigue and depression J Neurol Sci 2002 205 51 58 10.1016/S0022-510X(02)00312-X 12409184

[B31] D’Alisa S Miscio G Baudo S Simone A Tesio L Mauro A Depression is the main determinant of quality of life in multiple sclerosis: a classification-regression (CART) study Disabil Rehabil 2006 28 307 314 10.1080/09638280500191753 16492625

[B32] Fruehwald S Loeffler-Stastka H Eher R Saletu B Baumhackl U Depression and quality of life in multiple sclerosis Acta Neurol Scand 2001 104 257 261 11696017 10.1034/j.1600-0404.2001.00022.x

[B33] Kaminska M Kimoff R Benedetti A Robinson A Bar-Or A Lapierre Y Schwartzman K Trojan D Obstructive sleep apnea is associated with fatigue in multiple sclerosis Multiple Sclerosis Journal 2012 18 1159 1169 10.1177/1352458511432328 22183937

[B34] Kaminska M Kimoff RJ Schwartzman K Trojan DA Sleep disorders and fatigue in multiple sclerosis: Evidence for association and interaction J Neurol Sci 2011 302 7 13 10.1016/j.jns.2010.12.008 21241993

[B35] Veauthier C Paul F Fatigue in multiple sclerosis: which patient should be referred to a sleep specialist? Multiple Sclerosis Journal 2012 18 248 249 10.1177/1352458511411229 21652611

[B36] Veauthier C Radbruch H Gaede G Pfueller C Dörr J Bellmann-Strobl J Wernecke K-D Zipp F Paul F Sieb J Fatigue in multiple sclerosis is closely related to sleep disorders: a polysomnographic cross-sectional study Multiple Sclerosis Journal 2011 17 613 622 10.1177/1352458510393772 21278050

[B37] Fisk JD Brown MG Sketris IS Metz LM Murray TJ Stadnyk KJ A comparison of health utility measures for the evaluation of multiple sclerosis treatments J Neurol Neurosurg Psychiatry 2005 76 58 63 10.1136/jnnp.2003.017897 15607996 PMC1739294

[B38] Rudick RA Cutter G Baier M Fisher E Dougherty D Weinstock-Guttman B Mass MK Miller D Simonian NA Use of the Multiple Sclerosis Functional Composite to predict disability in relapsing MS Neurology 2001 56 1324 1330 10.1212/WNL.56.10.1324 11376182

[B39] Bock M Brandt AU Kuchenbecker J Dörr J Pfueller CF Weinges-Evers N Gaede G Zimmermann H Bellmann-Strobl J Ohlraun S Impairment of contrast visual acuity as a functional correlate of retinal nerve fibre layer thinning and total macular volume reduction in multiple sclerosis Br J Ophthalmol 2012 96 62 67 10.1136/bjo.2010.193581 21378002

[B40] Walter SD Ishikawa H Galetta KM Sakai RE Feller DJ Henderson SB Wilson JA Maguire MG Galetta SL Frohman E Ganglion cell koss in relation to visual disability in multiple sclerosis Ophthalmology 2012 119 1250 1257 10.1016/j.ophtha.2011.11.032 22365058 PMC3631566

[B41] Mowry EM Loguidice MJ Daniels AB Jacobs DA Markowitz CE Galetta SL Nano-Schiavi ML Cutter GR Maguire MG Balcer LJ Vision related quality of life in multiple sclerosis: correlation with new measures of low and high contrast letter acuity J Neurol Neurosurg Psychiatry 2009 80 767 772 10.1136/jnnp.2008.165449 19240050

[B42] Heesen C Böhm J Reich C Kasper J Goebel M Gold S Patient perception of bodily functions in multiple sclerosis: gait and visual function are the most valuable Mult Scler 2008 14 988 991 10.1177/1352458508088916 18505775

